# Use of Multiple Displacement Amplification as Pre-polymerase Chain Reaction (Pre-PCR) to amplify genomic DNA of siphonapterids preserved for long periods in scientific collections

**DOI:** 10.1186/1756-3305-3-86

**Published:** 2010-09-15

**Authors:** Daniel M Avelar, Pedro M Linardi

**Affiliations:** 1 Departamento de Parasitologia, Instituto de Ciências Biológicas da Universidade Federal de Minas Gerais, Caixa Postal 486, Avenida Antônio Carlos, 6627, Campus UFMG, Minas Gerais, 31270-901, Brazil

## Abstract

The recently developed Multiple Displacement Amplification technique (MDA) allows for the production of a large quantity of high quality genomic DNA from low amounts of the original DNA. The goal of this study was to evaluate the performance of the MDA technique to amplify genomic DNA of siphonapterids that have been stored for long periods in 70% ethanol at room temperature. We subjected each DNA sample to two different methodologies: (1) amplification of mitochondrial 16S sequences without MDA; (2) amplification of 16S after MDA. All the samples obtained from these procedures were then sequenced. Only 4 samples (15.4%) subjected to method 1 showed amplification. In contrast, the application of MDA (method 2) improved the performance substantially, with 24 samples (92.3%) showing amplification, with significant difference. Interestingly, one of the samples successfully amplified with this method was originally collected in 1909. All of the sequenced samples displayed satisfactory results in quality evaluations (Phred ≥ 20) and good similarities, as identified with the BLASTn tool. Our results demonstrate that the use of MDA may be an effective tool in molecular studies involving specimens of fleas that have traditionally been considered inadequately preserved for such purposes.

## Findings

The Neotropical region (excluding Mexico) contains 52 genera and about 280 species of fleas, and one of the most important families is Tungidae [1-3]. Females of the genus *Tunga *Jarocki, 1838 (Tungidae) penetrate into the skin of their hosts, including armadillos, anteaters, rodents, pigs, humans, dogs and other domestic animals [4]. After mating, gravid females undergoes hypertrophy, becoming a neosome 5-10 mm in size [5-7]. Taxonomic knowledge regarding siphonapterids is based primarily on morphological analysis of specimens permanently mounted on slides or preserved in 70% ethanol [3]. With the advent of the polymerase chain reaction (PCR) and other molecular approaches, DNA has become an excellent tool to study phylogenetic relationships [8]. However, in fleas few studies have analyzed genetic diversity in preserved specimens [9-11].

Many species of fleas have been identified from a small number of adult specimens mounted on permanent slides or adults and neosomes preserved in 70% ethanol at room temperature in scientific collections [12]. The DNA of samples preserved under these conditions for long periods is generally of poor quality and is often degraded, preventing its use in molecular studies [13]. Recently, some newly-developed methods have demonstrated high efficiency in the amplification of whole genomes from small amounts of template DNA. One of theses is the multiple displacement amplification method (MDA), a process that uses the bacteriophage φ29 DNA polymerase and exonuclease-resistant, thiophosphate-modified, degenerate hexamers to amplify genomic DNA from crude or pure sources and that has been used in some studies [14-19]. The enzyme φ29 DNA polymerase replicates DNA at a constant temperature of 30°C in a few hours without a thermal cycler and includes more complete genome coverage and unbiased amplification compared with PCR-based methods [14]. The goal of this study was to evaluate the use of MDA for the amplification of genomic DNA from siphonapterids preserved in scientific collections for long periods in 70% ethanol at room temperature.

Twenty-six of the samples analyzed were obtained from specimens deposited in the ectoparasites collection of the Department of Parasitology at the Universidade Federal de Minas Gerais and the Museum of Zoology at the Universidade de São Paulo. All of the samples were preserved in 70% ethanol at room temperature (Table 1). Samples used as controls [two *Ctenocephalides felis felis *(Bouché, 1835) and two *Tunga penetrans *(L., 1758)], were collected and preserved in absolute ethyl-alcohol and stored at - 20°C.

**Table 1:** Sampling data for the species analyzed.

Samples	Siphonapterids	Stages	Location	Hosts	Collection Date	Scientific collection^1^
1	*C. felis felis*	1 female	Belo Horizonte/MG	*Canis familiaris*	2008	UFMG
2	*C. felis felis*	1 female	Serra do Cipó/MG	*Leopardus pardalis*	1998	UFMG
3, 4	*C. felis felis*	2 females	Belo Horizonte/MG	*C. familiaris*	1988	UFMG
5, 6 and 7	*C. felis felis*	3 females	Ouro Preto/MG	*C. familiaris*	1979	UFMG
8 and 9	*T. penetrans*	1 female, 1 male	Vale do Mucuri/MG	___	2007	UFMG
10, 11 and 12	*T. penetrans*	3 neosomes	Brasília/DF	*Tamandua tetradactyla*	2002	UFMG
13	*T. caecata*	1 neosome	Santa Bárbara/MG	*Akodon cursor*	2007	UFMG
14	*T. caecata*	1 neosome	Nova Lima/MG	*Rhipidomys mastacalis*	2006	UFMG
15	*T. caecata*	1 neosome	Nova Lima/MG	*R. mastacalis*	2005	UFMG
16 and 17	*T. caecata*	2 neosomes	Curitiba/PR	*Rattus norvegicus*	1990	UFMG
18	*T. caecata*	1 neosome	Ouro Preto/MG	*R. rattus*	1989	UFMG
19	*T. caecata*	1 neosome	Caratinga/MG	*Nectomys squamipes*	1980	UFMG
20	*T. terasma*	1 female	Alegre/ES	*Dasypus novemcinctus*	2003	UFMG
21	*T. terasma*	1 neosome	Unaí/MG	*Priodontes maximus*	1996	UFMG
22	*T. travassosi*	1 neosome	Belo Horizonte/MG	*Dasypus novemcinctus*	1966	UFMG
23	*T. travassosi*	1 neosome	São Paulo/SP	*Dasypus novemcinctus*	1916	USP
24	*T. bondari*	1 neosome	Franca/SP	*Cariama cristata*	1909	USP
25 and 26	*Tunga *sp.	2 neosomes	Itatiaia/RJ	*Delomys dorsalis*	2000	UFMG

^1 ^Department of Parasitology of the Universidade Federal de Minas Gerais (UFMG) and Museum of Zoology of the Universidade de São Paulo (USP). All samples were preserved in 70% ethanol and stored at room temperature.

**Figure 1:**
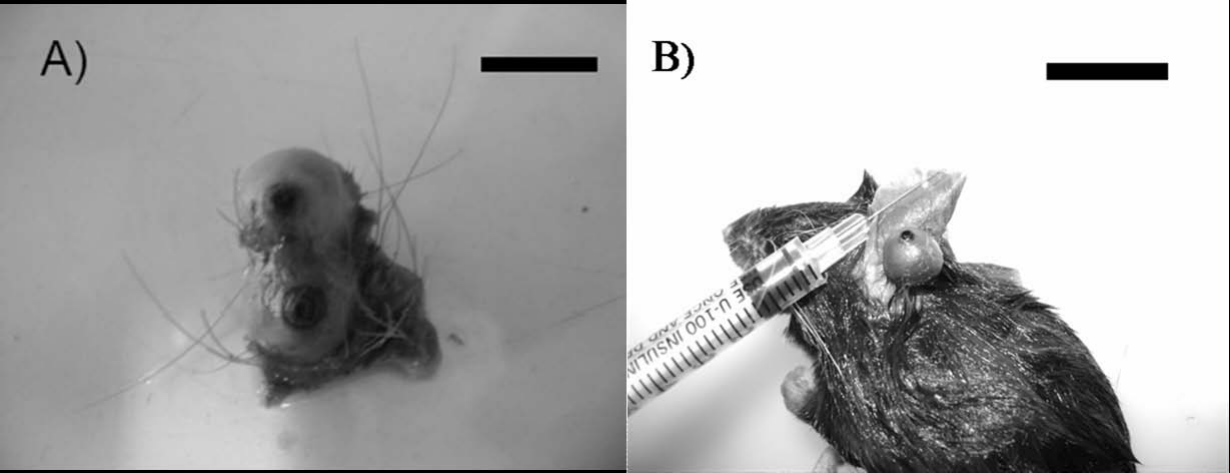
**Neosomes of some tungids**. A) *Tunga travassosi*, female. Bar: 8 mm B) *Tunga caecata*, female. Bar: 12 mm.

Genomic DNA was isolated using the DNeasy Tissue Kit (QIAGEN). Samples of adult fleas or neosomes (Figure 1) were used as the sources for DNA extraction. The specimens (a flea or a neosome) were dissected, mechanically shredded, and incubated with lysis buffer containing Proteinase K for 4 h. Afterwards, we followed the recommended protocols supplied by the manufacturer [10]. Following extraction, we subjected each sample to two different methodologies: (1) amplification of mitochondrial 16S sequences without MDA; (2) amplification of 16S after MDA. The exoskeletons obtained from the dissection were permanently mounted on slides [3]. The Repli-g^® ^Fast Mini kit (QIAGEN) was used for MDA of siphonapterid DNA, according to the manufacturer's instructions. The MDA-DNA preparations were stored at -20°C.

PCR amplification of DNA from single flea or neosome samples was performed using the primers Sen-mt16S TAC ATA ACA CGA GAA GAC C and Rev-mt16S GTG ATT GCG CTG TTA TCC, as previously described [20]. The HotStar MasterMix Kit was used for PCR reactions, according to the manufacturer's instructions (QIAGEN). The samples were scored as positive for amplification if a PCR product of approximately 200 bp was detected. No-template control reactions were routinely performed.

PCR products were purified using a QIAquick Gel Extraction Kit (QIAGEN). The BigDye^® ^Terminator v3.1 Cycle Sequencing Kit (APPLIED BIOSYSTEMS) was used for sequence determination by following the manufacturer's instructions. Sequencing was performed in an automatic sequencer (3130 × l APPLIED BIOSYSTEMS).

The sequences produced were analyzed using the CodonCode Aligner software (Phred algorithm) for the analysis of sequencing quality. Additionally, the samples obtained were submitted to the BLASTn tool from the National Center of Biotechnology Information (NCBI) to evaluate sequence similarity. The chi-square test with the Yates correction was used to compare the two methodologies: without or with MDA.

Morphological analysis identified the following species [3,21]: *Tunga penetrans, T. bondari *Wagner, 1932*, T. caecata *(Enderlein, 1901)*, T. terasma *Jordan, 1937*, T. travassosi *Pinto & Dreyfus, 1927*, Tunga *sp. and *C. felis felis *(Table 1).

**Figure 2:**
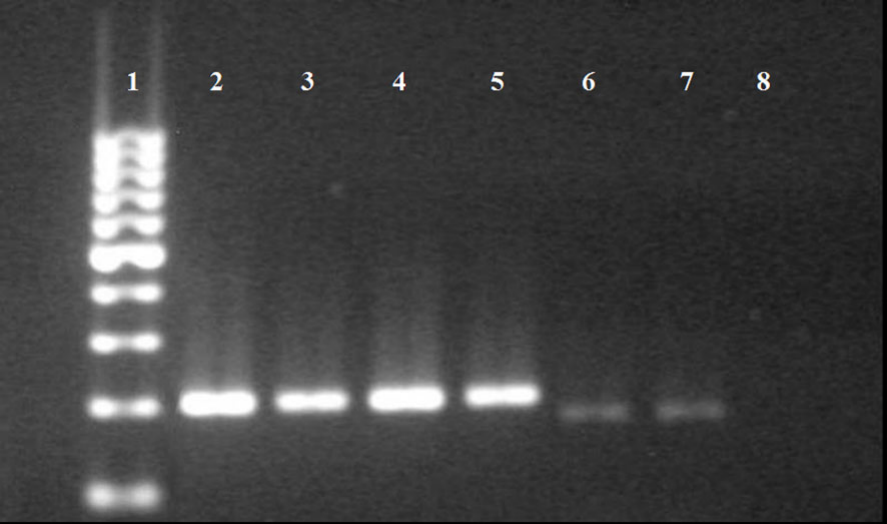
**Amplification, without the use of the MDA technique, of the 200 bp fragment of the 16S mtDNA of samples from scientific collections preserved in 70% ethanol and stored at room temperature**. 1) 100 bp DNA Marker; 2) *Tunga penetrans *(control); 3) *Ctenocephalides felis felis *(control); 4) *C. felis felis *(2008); 5) *T. penetrans *(2007); 6 and 7) *T. caecata *(2005 and 2007); 8) Negative control.

**Figure 3:**
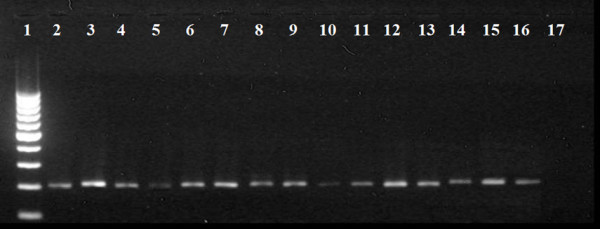
**Amplification, with the use of the MDA technique, of the 200 bp fragment of the 16S mtDNA gene of samples from scientific collections preserved in 70% ethanol and stored at room temperature**. 1) 100 bp DNA Marker; 2) *Tunga penetrans *(control); 3) *Ctenocephalides felis felis *(Control); 4 and 5) *T. penetrans *(2007 and 2002); 6 and 7) *T. travassosi *(1966 and 1916); 8, 9 and 10) *T. caecata *(2007, 1980 and 1989); 11 and 12) *T. terasma *(1996 and 2003); 13) *T. bondari *(1909); 14 and 15) *Tunga *sp. (2000); 16) *C. felis felis *(1988); 17) Negative control.

Without the use of MDA, only four samples (15.4%) displayed successful amplification of the 16s sequences (one of *C. felis felis*, collected in 2008; one of *T. penetrans*, collected in 2007 and two of *T. caecata*, collected in 2005 and 2007) (Figure 2). In contrast, application of the MDA technique permitted amplification from 24 samples (92.3%), presenting significant differences between the methods employed (χ^2 ^= 27.92; p < 0.01). These samples varied greatly in their collection times: *T. penetrans *(N = 5, in 2002 and 2007); *T. travassosi *(N = 2, in 1916 and 1966); *T. caecata *(N = 6, in 1970, 1980, 1990, 2005, 2006, 2007); *T. terasma *(N = 2, in 1996, 2003); *T. bondari *(N = 1, in 1909); *Tunga *sp. (N = 2, in 2000); *C. felis felis *(N = 6, in 1979, 1988, 1998, 2008) (Figure 3). Both amplification protocols (with and without the use of MDA) generated fragments of approximately 200 bp when performed on positive control samples.

**Figure 4:**
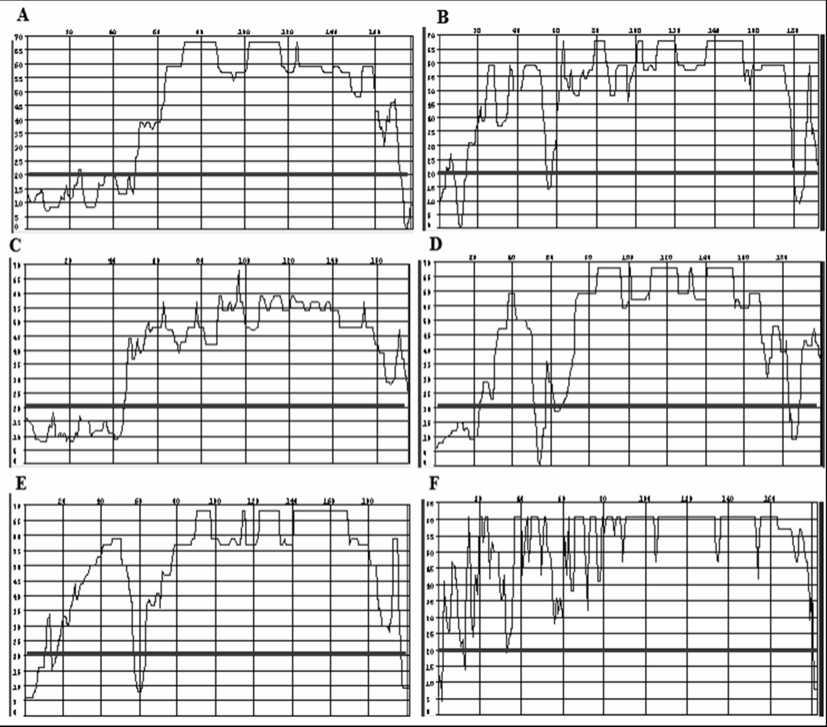
**Analysis of sequence quality of the 16S mtDNA gene fragment using the CodonCode Aligner software**. The black line denotes Phred = 20. A) *Tunga penetrans*; B) *Ctenocephalides felis felis; *C) *T. caecata*; D) *T. travassosi*; E) *Tunga *sp.; F) *T. terasma*.

**Figure 5:**
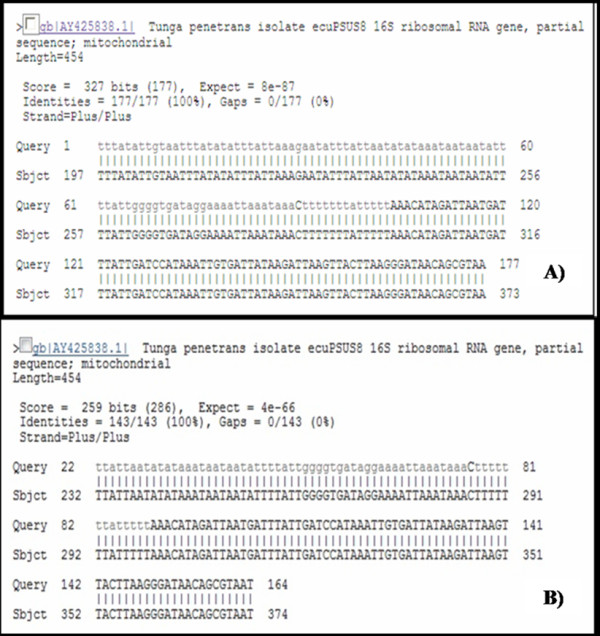
**Analysis of similarity between some of the sequences (177 pb) of the 16S mtDNA gene fragment from fleas preserved in scientific collections and sequences deposited in GenBank, as analyzed using the BLASTn tool**. A) *Tunga penetrans *collected in 2002; B) *T. bondari *collected in 1909.

All the sequences obtained showed satisfactory quality (Phred ≥ 20) (Figure 4). The samples of *T. penetrans *evaluated using the BLASTn tool showed a 100% degree of similarity with the reference sequence. The other samples did not have specific sequence references, but the primary matches identified were always to samples belonging to the same genus (Figure 5).

Collections are vital for taxonomic research and the study of biodiversity [22]. Otherwise, insects preserved in absolute or 70% ethanol and maintained at room temperature show a loss in the quality and quantity of DNA, compromising their use in genetic analysis [13]. Our results support this observation, as we were only able to amplify the 200 bp fragment corresponding to the 16S region of the mitochondrial rDNA from recently preserved samples.

In this study, we demonstrate the highly effective use of a commercially available MDA-based method for whole genome amplification of siphonapterid DNA from samples of adults and neosomes preserved in 70% ethanol at room temperature. The use of the MDA technique prior to the amplification of the target DNA (mtDNA 16S) allowed us to detect the 200 bp fragment in samples preserved under non-ideal conditions for long periods, including from one specimen collected in 1909. MDA-based commercial kits have been successfully used in the amplification of total genomic DNA from a wide range of organisms, including humans [23], dogs [17], pygmy elephantids [19], nematodes [18], mites [16] and insects [15]. A question raised by some authors is the possibility of amplification of contaminating DNA, instead of DNA from the target organism [18]. The similarity analysis performed using the BLASTn tool excluded the possibility of contamination, since the sequences obtained showed a 100% similarity to known samples (*T. penetrans*). For samples lacking reference sequences in the NCBI database, the primary matches identified were all from specimens belonging to the same genus, reinforcing the absence of contamination.

One of the possible applications of the MDA technique is its use in the amplification of DNA from samples with historical value that have been maintained in scientific collections [15,16,18]. The results obtained here support this idea, as this is the first reported use of the MDA technique in the amplification of DNA from fleas stored in museums. It is important to point out that, concerning tungid fleas, a large part of samples preserved in scientific collections is constituted by neosomes removed from several sites of their hosts (Figures 1, 2). Although some museums do not allow dissection of type specimens due to the risks of damage [24], sometimes it is necessary to dissect neosomes for identification, since that a dissected specimen is not a damaged specimen. Among siphonapterids, many species are morphologically indistinguishable (eg, some female fleas Rhopalopsyllidae) or have their identification difficult due to loss of structures (eg, loss of legs and bristles in Tungidae, during the formation of the neosome) [3,21]. Moreover, the morphological characteristics used in the identification of species of fleas are often autapomorphic, having limited use for phylogenetic reconstruction [11]. On that account, sometimes fleas need to be genotyped. Otherwise, fleas act as vectors of several diseases, including bacteria, Protozoa and helminths [25-27], in which the quantity of endosymbionts available for molecular analysis is often limited.

The combined application of MDA and conventional protocols for siphonapterid DNA amplification may be a valuable new tool for molecular studies concerning taxonomy, phylogeny and epidemiology, involving samples of fleas that have, until now, been considered inadequately preserved for such purposes.

## Competing interests

The authors declare that they have no competing interests.

## Authors' contributions

PML and DMA contributed equally to this work by performing all experiments and writing the manuscript.
